# Children with type 1 diabetes who experienced a honeymoon phase had significantly lower LDL cholesterol 5 years after diagnosis

**DOI:** 10.1371/journal.pone.0196912

**Published:** 2018-05-16

**Authors:** Benjamin Udoka Nwosu, Bo Zhang, Sanaa S. Ayyoub, Stephanie Choi, Tony R. Villalobos-Ortiz, Laura C. Alonso, Bruce A. Barton

**Affiliations:** 1 Division of Endocrinology, Department of Pediatrics, University of Massachusetts Medical School, Worcester, Massachusetts, United States of America; 2 Department of Quantitative Health Sciences, University of Massachusetts Medical School, Worcester, Massachusetts, United States of America; 3 Diabetes Division, Department of Internal Medicine, University of Massachusetts Medical School, Worcester, Massachusetts, United States of America; Baylor College of Medicine, UNITED STATES

## Abstract

**Importance:**

Landmark studies showed that partial clinical remission in new-onset type 1 diabetes is associated with reduced prevalence of long-term complications, but early clinical indicators of this favorable outcome are poorly characterized.

**Aim:**

To determine if there were any differences in lipid parameters, especially LDL-cholesterol, between remitters and non-remitters 4 to 5 years after the diagnosis of type 1 diabetes after controlling for hemoglobin A1c, body mass index, and pubertal status.

**Subjects and methods:**

A longitudinal retrospective cohort study of 123 subjects of mean age 11.9 ± 2.9 years, [male 11.7 ± 2.9 years, (n = 55); female 12.0 ± 2.9 years, (n = 68), p = 0.60] with type 1 diabetes of 4–5 years duration. Anthropometric and biochemical data were collected at the 4^th^ or 5^th^ year after diagnosis in line with the American Diabetes Association recommendation to initiate screening for complications in children either at the beginning of puberty or 4–5 years after diagnosis. Puberty was defined by Tanner stages II-V. Partial clinical remission was defined by the gold-standard insulin-dose adjusted hemoglobin A1c (IDAA1c) of ≤9.

**Results:**

There were 44 (35.8%) remitters (age 13.0 ± 2.5y; male 52.3%). Both the total cholesterol and LDL-cholesterol were significantly lower in remitters compared to non-remitters: LDL-C: 78.8 ± 28.7 mg/dL vs. 91.6 ± 26.5 mg/dL, p = 0.023; and total cholesterol: 151.5 ± 32.6 mg/dL vs. 167.0 ± 29.6 mg/dL, p = 0.015. Other lipid fractions were similar between the groups. There were no differences between the groups for glycemic control, body mass index z score, thyroid function, celiac disease occurrence, or vitamin D status. A greater number of remitters were in puberty compared to non-remitters (86.4% vs. 60.8%, p = 0.006). LDL-C concentration was similar in prepubertal remitters vs. non-remitters (p = 0.93), but was significantly lower in remitters in puberty compared to non-remitters in puberty (p = 0.018) after adjusting for age and duration of diabetes.

**Conclusions:**

Children with type 1 diabetes who underwent a honeymoon phase had significantly lower LDL cholesterol 5 years after diagnosis. This early divergence in lipidemia may explain the dichotomy in the prevalence of long-term complication in type 1 diabetes between remitters and non-remitters. It also offers a pathway for targeted lipid monitoring in type 1 diabetes, by establishing non-remission as a non-modifiable risk factor for vascular complication in type 1 diabetes.

## Introduction

Landmark studies showed that partial clinical remission (PCR), also known as the honeymoon phase, in new-onset type 1 diabetes is associated with reduced prevalence of long-term complications of type 1 diabetes [1, 2]; however, the early clinical indicators of this outcome in the early phase of type 1 diabetes are poorly characterized. The recently published scientific statement by the Endocrine Society on Diabetic Microvascular Disease noted that vascular complications are the major cause of mortality and morbidity in patients with diabetes[3] but the role of PCR on cardiovascular outcome in patients with type 1 diabetes was not fully assessed in that statement. This is crucial as >50% of children and adolescents with new-onset type 1 diabetes fail to undergo PCR[4–7]. These non-remitters have poorer short- and long-term diabetes outcomes compared to those who experienced PCR, also known as remitters[1, 8–10]. This dichotomy in outcome was recently demonstrated in a longitudinal study in young adults that found a significantly reduced risk for chronic microvascular complications at 7-year follow-up in patients who experienced PCR[11]. Thus, patients who experienced PCR have an overall prognostic advantage over non-remitters.

However, the basic pathobiology of the early-phase vasculopathy in children with type 1 diabetes is not fully known. Though the Diabetes Control and Complications Trial reported a protective role for C-peptide on vasculature in remitters[2], data are lacking on the characterization of early dyslipidemia in both remitters and non-remitters; and more importantly, whether early-phase dyslipidemia could explain the dichotomy in the presentation of long-term vascular complications in type 1 diabetes based on remission history. A longitudinal study in children reported that approximately 25% of youth with type 1 diabetes have progressive and persistent dyslipidemia[12], and children and adolescents with type 1 diabetes have increased arterial stiffness, a marker of atherosclerosis, compared to healthy controls[13]. Therefore, the investigation of the clinical indicators of early dyslipidemia in children with type 1 diabetes becomes crucial as cardiovascular disease is the leading cause of death in adults with type 1 diabetes [14, 15]. More importantly, the atherosclerotic process starts in childhood and early adolescence[16, 17], and dyslipidemia is a major contributor to the risk for cardiovascular disease[16, 17].

Earlier studies have attributed the dyslipidemia in type 1 diabetes to increased weight gain and worsening of glycemic control[12, 18], but a detailed characterization of early-phase dyslipidemia of type 1 diabetes based on stratification of patients by PCR history has not been previously done. Such an investigation would clarify the possible role of non-remission as a non-modifiable risk factor for dyslipidemia in type 1 diabetes.

The aim of this study, therefore, was to determine if there were any significant differences in LDL-cholesterol between remitters and non-remitters 4–5 years after the diagnosis of type 1 diabetes after controlling for hemoglobin A1c (HbA1c) and body mass index (BMI). The study’s hypothesis was that serum LDL-C concentration would be similar between remitters and non-remitters in the first few years after the diagnosis of type 1 diabetes. LDL-C was chosen as the primary outcome because it is the primary lipid marker of cardiovascular disease risk in both adolescents and adults with type 1 diabetes [17, 19]; and has been jointly endorsed for cardiovascular risk assessment in youth by the Integrated Guidelines of Cardiovascular Health and Risk Reduction in Children and Adolescents and the American Diabetes Association[20, 21]. The timeline of 4–5 years after diagnosis was chosen because it is in line with the American Diabetes Association recommendation to initiate screening for complications of diabetes in children either at the inception of puberty or 4–5 years after diagnosis[21].

## Subjects and methods

### Ethics statement

The Institutional Review Board of the University of Massachusetts approved the study protocol. The medical records of all subjects were anonymized and de-identified prior to analysis.

### Subjects

The patient population included 123 pediatric patients with a confirmed diagnosis of type 1 diabetes from the Children’s Medical Center Database of the UMassMemorial Medical Center, Worcester, Massachusetts, USA. As described in detail previously[4, 22], we established the diagnosis of type 1 diabetes based on any of the following glycemic indices: a fasting blood glucose of ≥ 126 mg/dL (7 mmol/L), and/or 2-hour postprandial glucose of ≥200 mg/dL (11.1 mmol/L), and/or random blood glucose of ≥200 mg/dL with symptoms of polyuria and/or polydipsia, in addition to the detection of one or more diabetes-associated auto-antibodies, namely glutamic acid decarboxylase antibodies, islet cell cytoplasmic autoantibodies, insulin autoantibodies, and insulinoma-associated-2 autoantibodies. Patients with other forms of diabetes mellitus were excluded from the study.

As previously reported[4, 5], following the diagnosis of type 1 diabetes mellitus, children that were not in diabetic ketoacidosis were started on a standard basal-bolus insulin regimen, consisting of injections of once-daily long-acting insulin and pre-meal short-acting insulin. Patients in diabetic ketoacidosis were started on an insulin drip at 0.05 units/kg/hour. The insulin drip rate was titrated to maintain euglycemia until the resolution of acidosis. All patients were discharged from the hospital on a basal-bolus insulin regimen.

We have previously published that data collection for anthropometric, clinical, and biochemical parameters were conducted at the time of diagnosis, and then every 3 months for the first year, and every 3 to 6 months until 36 months[4, 5]. For this study, anthropometric and biochemical data were collected at the 4^th^ year or 5^th^ year visit in line with the American Diabetes Association recommendation for the initiation of screening for diabetes complication in children with type 1 diabetes either at the inception of puberty or 4–5 years after diagnosis[21]. PCR was defined by insulin dose-adjusted hemoglobin A1c (IDAA1c) of ≤9[23], as well as by a total daily dose of insulin (TDD) of <0.3 units/kg/day, which was recently validated by our group to be highly correlated with IDAA1c[4]. The IDAA1c, which integrates HbA1c and TDD, is considered the gold standard parameter for the detection of PCR[23]. It has been validated in multiple cohort studies[24, 25] and is useful for the characterization of PCR in clinical studies. The formula for IDAA1C is HbA1c (%) + [4 X total daily dose of insulin (units/kg/24h)][23].

### Anthropometry

The procedure for anthropometry has been previously described in detail[4, 5, 26]. Weight was measured to the nearest 0.1 kg using an upright scale. Height was measured to the nearest 0.1 cm using a wall-mounted stadiometer that was calibrated daily. BMI was calculated from the formula: weight/height^2^ (kg/m^2^), and expressed as standard deviation score (SDS) for age and sex, based on National Center for Health Statistics (NCHS) data[27]. Overweight was defined as BMI of ≥85^th^ but <95^th^ percentile, and obesity was defined as BMI of ≥95^th^ percentile for age and gender. Sexual maturity rating was denoted by Tanner staging with puberty marked by Tanner II-V stages.

### Assays

The assay methodologies have been previously described[4, 26, 28]. Whole blood sample was collected for the estimation of HbA1c; and serum was collected for the estimation of other analytes. Hemoglobin A1c was measured by DCA 2000+ Analyzer (Bayer, Inc., Tarrytown, NY, USA) based on Diabetes Control and Complications Trial standards [29]. Serum 25-hydroxyvitamin D [25(OH)D] concentration was analyzed using 25-hydroxy chemiluminescent immunoassay (DiaSorin Liaison; Stillwater, Minnesota). Serum lipids were measured at the University of Massachusetts Medical School Clinical Laboratory based on the Beckman Coulter AU system which has been certified to meet the National Cholesterol Education Program’s criteria for accuracy[30]. When triglycerides were ≥400 mg/dL, LDL-C was either measured by the beta quantification procedure or calculated by the Friedwald equation[31]. Diabetes-associated autoantibodies were measured by Quest Diagnostics, Chantilly, VA, USA. GAD-65 assay was performed using enzyme-linked immunosorbent assay, and IA-2A and IAA assays were performed using radio-binding assay.

### Statistical analyses

As described in detail previously[4, 5], means and standard deviations (SD) were calculated for the continuous descriptive summary statistics and biochemical parameters. Two-sided Student’s t test was used to compare the two groups, remitters and non-remitters, as defined by both IDAA1c≤9 criterion (Table 1) and by TDD of <0.3 units/kg/day criterion respectively. Proportions were calculated for the presence of overweight or obesity (BMI >85th percentile), celiac disease antibody, anti-thyroid antibodies, microalbuminuria, puberty, and psychiatric illness. Comparison of binary variables between the two groups were performed using Pearson’s chi-squared test. Non-parametric variables were log transformed for analysis and later back transformed for reporting. The duration of PCR was calculated independently for each definition of PCR as the interval between the first and last documented time points with IDAA1C value of ≤9; as well as the first and last documented time points with TDD of <0.3 units/kg/day. All statistical analyses were performed by using R (version 3.4.2), an open source programming language and software environment for statistical computing and graphics (R Foundation for Statistical Computing, Vienna, Austria).

**Table 1:** Comparison of the anthropometric and biochemical characteristics of remitters and non-remitters at 4–5 years after the diagnosis of type 1 diabetes. Partial clinical remission was defined as an insulin-dose adjusted hemoglobin A1c of ≤9.

Parameters	Remitters (n = 44)	Non-Remitters (n = 79)	p-value
Age (year)	13.0 ± 2.5	11.3 ± 2.9	**<0.001**
Sex (male; female)	0.5 ± 0.5	0.4 ± 0.5	0.29
Height z-score	0.06 ± 1.0	-0.006 ± 1.2	0.76
Weight z-score	0.7 ± 0.8	0.5 ± 1.0	0.31
BMI z-score	0.7 ± 0.8	0.6 ± 0.9	0.59
Systolic Blood pressure (mm Hg)	111.3 ± 12.8	107.8 ± 11.8	0.13
Diastolic Blood pressure (mm Hg)	70.6 ± 6.0	70.2 ± 7.0	0.70
Proportion with BMI >85th percentile (%)	34.1	29.1	0.71
Proportion in puberty (Tanner II-V) (%)	86.4	60.8	**0.006**
Duration of type 1 diabetes	4.8 ± 0.4	4.8 ± 0.4	1.00
TDD (Units/kg/day) at 4–5 years	0.9 ± 0.4	1.0 ± 0.4	0.24
25-hydroxyvitamin D (ng/mL)	25.6 ± 10.0	28.3 ± 10.5	0.18
Hemoglobin A1c (mmol/mol)	70.4 ± 16.9	72.3 ± 13.5	0.53
Hemoglobin A1c (%)	8.6 ± 1.5	8.8 ± 1.2	0.53
Total cholesterol (TC) (mg/dL)	151.5 ± 32.6	167.0 ± 29.5	**0.015**
Triglycerides (mg/dL)	99.1 ± 65.7	99.6 ± 80.8	1.00
HDL-cholesterol (mg/dL)	53.2 ± 11.7	57.1 ± 14.3	0.12
LDL-cholesterol (mg/dL)	78.825	91.6 ± 26.5	**0.023**
TC/HDL	2.9 ± 0.7	3.8 ± 5.4	0.17
LDL/HDL	1.5 ± 0.6	1.7 ± 0.7	0.28
TG/HDL	1.1 ± 0.2	1.1 ± 0.2	0.74
Thyroid stimulating hormone (uIU/mL)	2.0 ± 1.3	2.1 ± 1.5	0.61
Seasons of vitamin D draw: (%) winter-spring	47.7	46.8	1.00
Proportion with positive celiac autoantibodies (TGAb) (%)	15.9	15.2	1.00
Proportion with positive anti-thyroid antibodies (TPOAb and TgAb) (%)	23.3	29.9	0.57
Proportion with microalbuminuria (ACR of > 30 μg albumin/mg creatinine) (%)	9.1	6.3	0.84
Proportion with psychiatric illness (%)	27.3	19	0.40

TDD total daily dose of insulin; TGAb tissue transglutaminase antibody; TPOAb thyroperoxidase antibody; TgAb thyroglobulin antibody; ACR albumin-creatinine ratio; high-density lipoproteins; LDL low-density lipoproteins; TG triglycerides. Significant *p* values are bolded.

## Results

### Anthropometry

This longitudinal retrospective cohort study analyzed the data of 123 subjects with type 1 diabetes of 4–5 years duration. Their mean age was 11.9 ± 2.9yr, [male 11.7± 2.9yr, (n = 55); female 12.0 ± 2.9yr, (n = 68), p = 0.60]. There were 44 (35.8%) remitters (age 13.0 ± 2.5yr; male 52.3%) as defined by IDAA1c ≤9 criterion; and 55 (44.7%) remitters (age 11.9 ± 2.9yr; male 49.1%) as defined by TDD of <0.3 units/kg/day criterion (p = 0.153). The remitters were older than the non-remitters; and a significant proportion of the remitters were in puberty compared to the non-remitters. There were no differences between the groups for BMI z score, blood pressure, or the proportion of overweight or obese patients (Table 1). When PCR was defined by TDD of <0.3 units/kg/day there was no significant difference in age between the remitters and non-remitters, 11.9 ± 2.9 years vs. 11.8 ± 3.1 years, p = 0.91. There was equally no difference in the proportion of male subjects between the remitters and non-remitters, 49.1% vs. 41.2%, p = 0.50; as well as in BMI z score, 0.56 ± 0.8 vs. 0.74 ± 0.9, p = 0.25.

### Biochemical parameters

When PCR was defined by IDAA1c of ≤9, both the total cholesterol (TC) and LDL-C were significantly lower in remitters compared to non-remitters: LDL-C: 78.8 ± 28.7 mg/dL vs. 91.6 ± 26.5 mg/dL, p = 0.023, and TC 151.5 ± 32.6 mg/dL vs. 167.0 ± 29.6 mg/dL, p = 0.015 (Table 1). Similarly, when PCR was defined by TDD<0.3 units/kg/day LDL-C was also significantly lower in remitters compared to non-remitters: 81.2 ± 27.8 mg/dL vs. 91.7 ± 27.3 mg/dL, p = 0.04. Other lipid fractions were similar between the groups by both definitions, and there were no differences between the groups for glycemic control as marked by HbA1c and TDD of insulin; vitamin D status as marked by serum 25(OH)D; BMI z score, thyroid stimulating hormone, and the proportion of subjects with elevated albumin-creatinine ratio of >30 μg albumin/mg creatine, autoimmune thyroid disease, celiac disease, or psychiatric illness.

**Fig 1:**
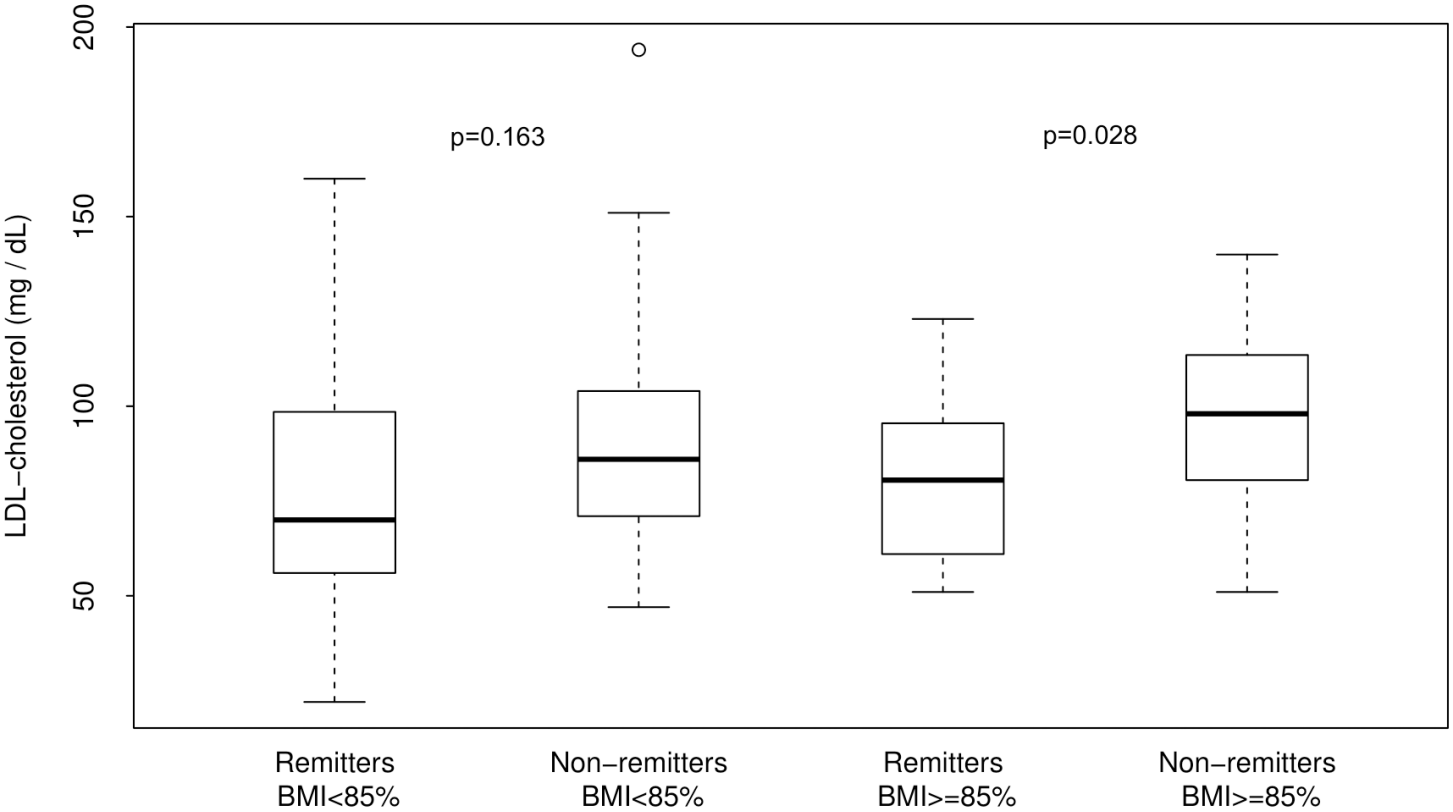
Analysis of the changes in serum LDL-cholesterol concentration in the first 4–5 years of type 1 diabetes stratified by both body mass index and remission status. Serum LDL-C was similar between the normal-weight remitters and non-remitters: 79.0 ± 32.8 mg/dL vs. 89.8 ± 27.5 mg/dL, p = 0.17. In contrast, LDL-C was significantly lower in remitters among the overweight/obese cohort, 78.5 ± 21.1 mg/dL vs. 95.6 ± 24.2 mg/dL, p = 0.028.

In subsequent analysis, subjects were stratified by BMI percentiles into normal-weight, as denoted by BMI of <85^th^ percentile, and overweight or obese, as denoted by BMI of ≥85^th^ percentile (Fig 1). There was no significant difference in LDL-C between the normal-weight remitters and non-remitters: 79.0 ± 32.8 mg/dL vs. 89.8 ± 27.5, p = 0.163. In contrast, LDL-C was significantly lower in remitters among the overweight/obese cohort by both definitions of PCR: IDAA1c, 78.5 ± 21.1 mg/dL vs. 95.6 ± 24.2, p = 0.028 (Fig 1); and by TDD <0.3 units/kg/day, 72.3 ± 21.4 mg/dL vs. 96.6 ± 21.8 mg/dL, p = 0.005, suggesting the possibility that early normoglycemia of PCR protected the subjects from developing early-phase dyslipidemia. However, since LDL-C was not measured at diagnosis, it is also unclear whether higher LDL-C at diagnosis predisposed subjects to non-remission.

**Fig 2:**
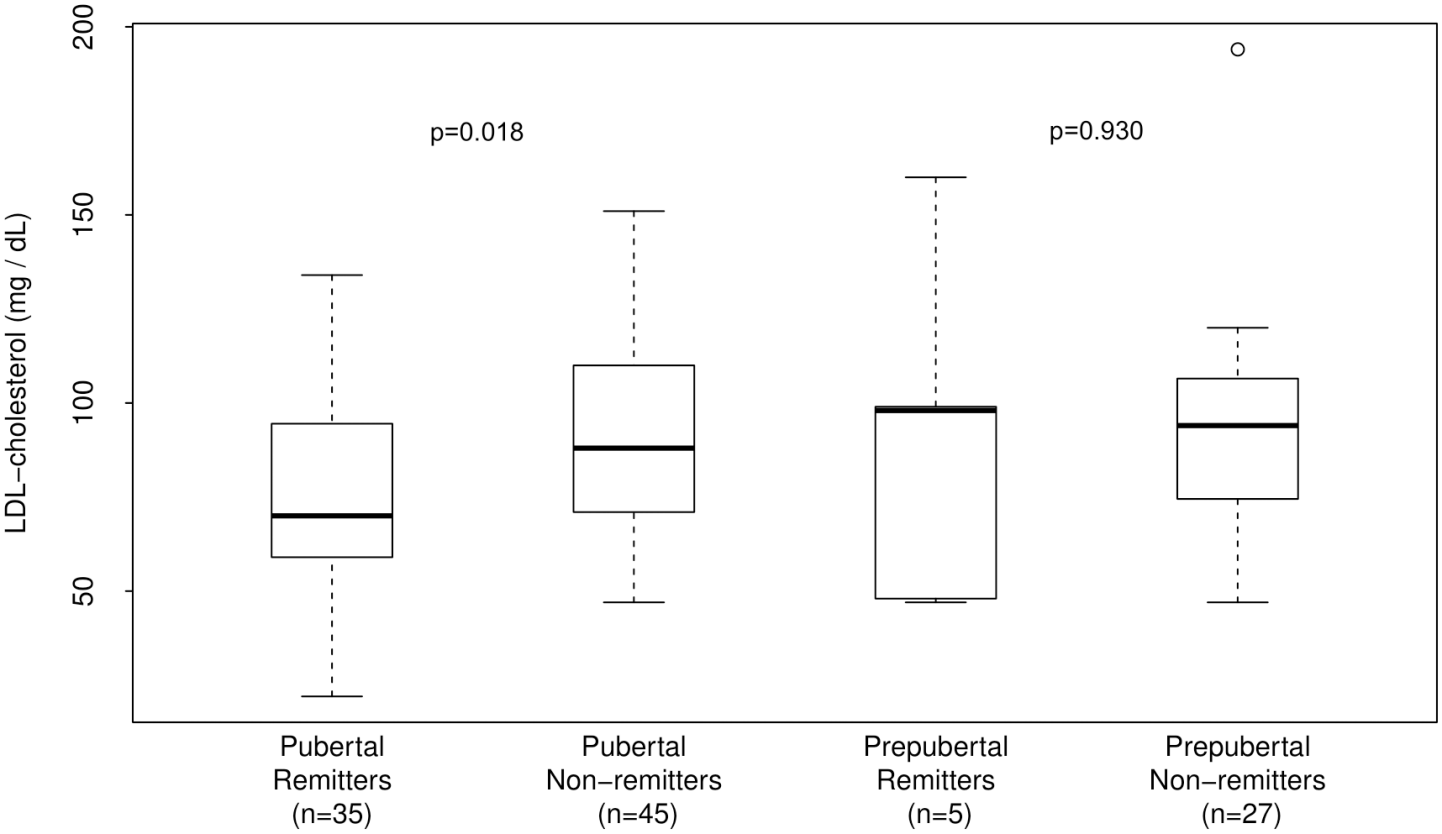
Analysis of the changes in serum LDL-cholesterol concentration in the first 4–5 years of type 1 diabetes stratified by both pubertal status and remission status. LDL-C concentration was similar in prepubertal remitters vs. non-remitters (p = 0.93), but was significantly lower in remitters in puberty compared to non-remitters in puberty (p = 0.018) after adjusting for age and duration of diabetes.

The role of age, duration of diabetes, and puberty (Fig 2) on the differences in LDL and TC between the groups was explored in subsequent analysis. The results showed that LDL-cholesterol was significantly lower in remitters in puberty compared to non-remitters in puberty after adjusting for age and duration of diabetes: 77.2 ± 25.8 mg/dL vs. 91.1 ± 25.6 mg/dL, p = 0.018. In contrast, there was no significant difference in LDL-C concentration between the prepubertal remitters and prepubertal non-remitters after adjusting for age and duration of diabetes: 90.4 ± 46.5 mg/dL vs. 92.4 ± 28.5 mg/dL, p = 0.93.

Similarly, TC was significantly lower in remitters in puberty compared to non-remitters in puberty after adjusting for age and duration of diabetes: 149.8 ± 32.1mg/dL vs. 168.0 ± 30.2 mg/dL, p = 0.0115. In contrast, there was no significant difference in TC concentration between the prepubertal remitters and prepubertal non-remitters: 163.4 ± 37.3 mg/dL vs. 165.3 ± 28.8 mg/dL, p = 0.92.

**Fig 3:**
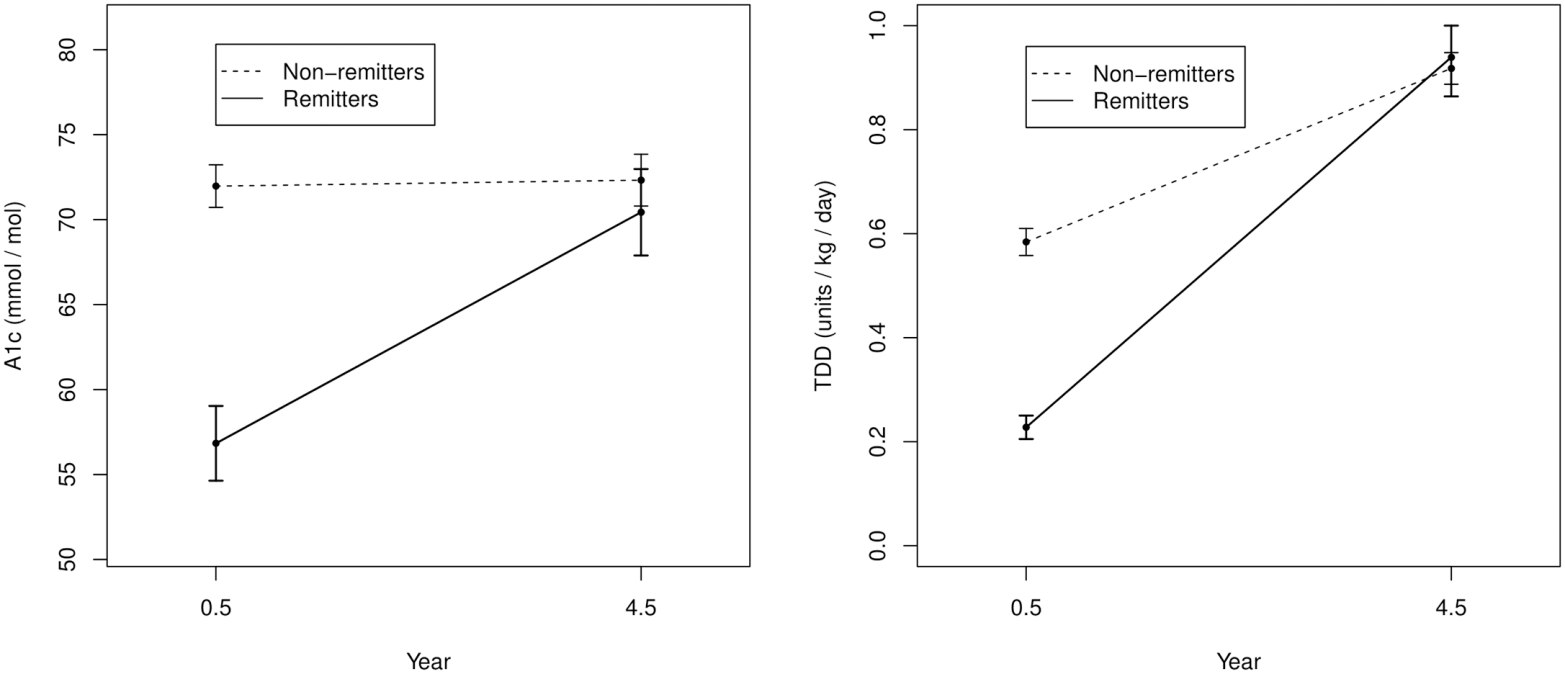
Longitudinal assessment of glycemic control in remitters and non-remitters based on the comparison of changes in (a) hemoglobin A1c and (b) total daily dose of insulin at 6 months and at 4–5 years following the diagnosis of type 1 diabetes. Both the HbA1c and Total daily dose (TDD) of insulin were significantly lower in the remitters at the peak of partial clinical remission (PCR) at 6 months compared to the subsequent values at 4–5 years: TDD, 0.22 ± 0.2 units/kg/day vs. 0.64 ± 0.6 units/kg/day, p<0.001; HbA1c 7.35 ± 1.3% vs. 8.6 ± 1.5%, p = 0.0001 (56.8 ± 14.6 mmol/mol vs. 70.4 ± 16.9 mmol/mol, p = 0.0001). In contrast, there was no difference in HbA1c between 6 months and 4–5 years among the non-remitters; 8.74 ± 1.0% vs. 8.77 ± 1.2, p = 0.57, (72.0 ± 11.1 mmol/mol vs. 72.3 ± 13.5 mmol/mol, p = 0.57).

To further investigate the role of early and later glycemic control on the LDL findings, glycemic control (marked by HbA1c and TDD of insulin) at 4–5 years was compared to similar glycemic control at the peak period of PCR at 6 month as detailed in our prior studies[4, 5]. The results, depicted in Fig 3, showed that both the TDD and HbA1c were significantly lower in the remitters at the peak of PCR at 6 months compared to the subsequent values at 4–5 years: TDD, 0.22 ± 0.17 units/kg/day vs. 0.64 ± 0.6 units/kg/day, p<0.001; HbA1c 7.35 ± 1.33% vs. 8.6 ± 1.5%, p = 0.0001 (56.8 ± 14.6 mmol/mol vs. 70.4 ± 16.9 mmol/mol, p = 0.0001). In contrast, among the non-remitters, there was no difference in HbA1c between 6 months and 4–5 years; 8.74 ± 1.0% vs. 8.77 ± 1.2%, p = 0.57, (72.0 ± 11.1 mmol/mol vs 72.3 ± 13.5 mmol/mol, p = 0.57). Interestingly, there was a significant increase in the TDD of insulin in non-remitters between 6 months and 4–5 years: 0.58 ± 0.21 vs 0.92 ± 0.25, p <0.001.

## Discussion

This study reports that children and adolescents who underwent PCR had significantly lower mean LDL cholesterol 4–5 years after the diagnosis of type 1 diabetes compared to their peers who did not experience PCR. This significantly lower LDL concentration in remitters was independent of age, glycemic control, adiposity, thyroid function, celiac disease occurrence, or vitamin D status. This significantly lower LDL in remitters is impressive given that a greater proportion of the remitters were in puberty 4–5 years after the diagnosis of type 1 diabetes compared to the non-remitters, and previous studies have reported that LDL-C is increased in youth with type 1 diabetes compared to healthy peers as children with type 1 diabetes do not show the usual pattern of decreasing LDL-C during puberty[32]. This study clarifies the above earlier report by demonstrating a significantly lower LDL-C concentration in remitters in puberty compared to non-remitters during puberty. In order words, remitters demonstrate similar LDL-C profile during puberty as healthy peers without type 1 diabetes.

For this study, PCR was principally defined by the gold standard parameter of an IDAA1c of ≤9[23]. An intriguing finding was that when subjects were stratified by BMI status, there was no difference in serum LDL-C concentration between the normal-weight subjects in both groups; however, among the overweight/obese subjects, LDL-C was significantly lower in the remitters compared to non-remitters. Equally, both LDL-C and TC were similar between prepubertal remitters vs. non-remitters, but were significantly lower in remitters in puberty compared to non-remitters in puberty.

Previous reports on dyslipidemia in pediatric type 1 diabetes may have been confounded by the lack of stratification of subjects by PCR history[12, 18, 33, 34]. One longitudinal study showed that about 25% children and adolescents with type 1 diabetes have abnormal and progressive dyslipidemia marked by similar and parallel increases in LDL-C and non-HDL cholesterol[12], while another study in youth with poorly-controlled type 1 diabetes reported a positive association between increased arterial stiffness and HbA1c, total cholesterol, and LDL-C[33]. But a longitudinal retrospective cohort study in youth with type 1 diabetes reported that changes in HbA1c and BMI z scores had only a modest impact on LDL and non-HDL cholesterol[18]. While some studies have reported a significant relationship between poor glycemic control and dyslipidemia in subjects with type 1 diabetes, others found inconsistent pattern of correlation of lipids and HbA1c[35], or no correlation[32]. This has prompted the recent Scientific Statement on vascular complications in subjects with diabetes mellitus to note that persistent hyperglycemia alone was not enough to explain vasculopathy in subject with diabetes mellitus; rather that yet unidentified genetic and endogenous protective factors play some role[3]. Unfortunately, none of the above studies examined the differences in lipid profiles based on the remission status of these youth with type 1 diabetes. This is of fundamental significance as it is unclear what proportion of these youth were either remitters or non-remitters, as this could have accounted for the reported inconsistencies in lipid outcomes. For example, the study that reported only a modest effect of HbA1c and BMI on lipid parameters[18] could have contained a higher proportion of remitters, while the study that reported progressive and persistent dyslipidemia in their cohort[12] could have contained a higher proportion of non-remitters. Therefore, it is crucial to stratify subjects based on PCR history in future investigations in lipidology in type 1 diabetes given that >50% of children and adolescents with new-onset type 1 diabetes do not experience PCR[6, 7], and that these non-remitters have an increased risk for complications[1, 8–10].

The exact mechanism of this increased risk is not fully known; however, three theories have been proposed to explain the beneficial effect of PCR on diabetes outcomes. The recent report of a significantly reduced risk for chronic microvascular complications at 7-year follow-up in young adult patients who experienced PCR [11] suggests that persistent hyperglycemia in the early phase of type 1 diabetes may lead to endothelial dysfunction and vascular disease in non-remitters. Our findings are consistent with the above report as the longitudinal assessment of glycemic control showed that the principal difference between the remitters and non-remitters was the period of early normoglycemia in remitters. The concept of the ‘hyperglycemic memory’ phenomenon partly explains this hyperglycemic effect, and proposes that the risk of the long-term complications of type 1 diabetes[11] could be reduced by prompt correction of persistent hyperglycemia in the early phase of type 1 diabetes.

A second potential mechanism is based on the protective role for C-peptide on the vasculature in patients with type 1 diabetes who have residual β-cell function[36]. The protective role on vasculature has led to the consideration of the use of exogenous C-peptide molecule in non-remitters for the prevention of vascular complications of diabetes mellitus[11].

A third potential mechanism that links dyslipidemia, glycemic control, and adiposity in type 1 diabetes is insulin resistance[12], a phenomenon that has been described in type 1 diabetes [37] owing to the rising prevalence of obesity, sedentary lifestyle, and chronic exogenous insulin administration[12]. Insulin resistance has been associated with atherogenic lipid profile in both diabetic- and non-diabetic patients[38]. In patients with diabetes, insulin resistance affects all lipoprotein fractions leading to the accumulation of chylomicrons and VLDL, while the enrichment of HDL and LDL by accumulating triglycerides lead to high levels of atherogenic particles and low levels of HDL cholesterol.

This study advances the field by clarifying that PCR is associated with lower likelihood of early dyslipidemia in type 1 diabetes at 4–5 years following the diagnosis of the disease. Though the exact mechanism for this favorable LDL-C profile by PCR is not known, our findings suggest the possibility that the hallmarks of PCR such as early euglycemia and reduced insulin resistance might protect against later dyslipidemia in youth who underwent PCR. It is unclear whether a tight glycemic control and the maintenance of normal-weight status in the first few years of diagnosis of type 1 diabetes would result in a similar reduction in LDL-C. On the other hand, it is possible that LDL-C might have also been lower in remitters at the onset of T1D, and that higher LDL-C might prevent PCR. Unfortunately, we do not have LDL-C measurements at the onset of T1D in our cohort. It is also possible that an upstream unmeasured genetic or other risk factor influences risk of both PCR and dyslipidemia. Regardless of the mechanism behind the association, it is our belief that the adoption of a simpler definition of PCR such as TTD <0.3 units/kg/day[4] would enable an early identification of non-remitters, and in turn, the institution of targeted therapeutic regimens to prevent early dyslipidemia in children and adolescents with type 1 diabetes.

Some of the limitations of this study include its retrospective design which precludes any causality between the parameters studied. We did not have data on serum C-peptide which limited our ability to test the reliability of the definition of PCR based on either IDAA1c or TDD. We also did not have data on insulin resistance which could have been correlated with glycemic excursions during and after PCR in remitters. However, this and our earlier reports show significantly lower HbA1c and insulin requirements in remitters during the early phase of type 1 diabetes[4]. Strengths of this study include the use of a representative sample of children with type 1 diabetes with respect to age, sex, race, BMI, and pubertal status; the extended period of data collection; evaluation of other disease conditions that could affect lipid profile; and the definition of PCR using IDAA1c criterion, with a secondary confirmation using TDD of <0.3 units/kg/day. These measures allowed for meaningful comparison of the outcomes between the remitters and non-remitters.

## Conclusions

Children who underwent PCR had significantly lower LDL cholesterol 5 years after their diagnosis with type 1 diabetes. This finding may explain the dichotomy in the prevalence of long-term complication in type 1 diabetes between remitters and non-remitters. It also offers a pathway for targeted lipid monitoring in type 1 diabetes, by establishing non-remission as a non-modifiable risk factor for vascular complication in type 1 diabetes. Additionally, the early divergence in lipid profile in these children suggests the need to modify the current American Diabetes Association guidelines to allow for the initiation of the assessment of lipid profile at the time of diagnosis to ensure early identification of dyslipidemia in the non-remitters. These measures will help to reduce the high prevalence of long-term complications in patients with type 1 diabetes.
